# Investigating the Impact of Delivery Routes for Exon Skipping Therapies in the CNS of DMD Mouse Models

**DOI:** 10.3390/cells12060908

**Published:** 2023-03-15

**Authors:** Amel Saoudi, Claire Fergus, Talia Gileadi, Federica Montanaro, Jennifer E. Morgan, Vincent P. Kelly, Thomas Tensorer, Luis Garcia, Cyrille Vaillend, Francesco Muntoni, Aurélie Goyenvalle

**Affiliations:** 1 Université Paris-Saclay, UVSQ, Inserm, END-ICAP, 78000 Versailles, France; 2 Université Paris-Saclay, CNRS, Institut des Neurosciences Paris-Saclay, 91400 Saclay, France; 3 School of Biochemistry & Immunology, Trinity Biomedical Sciences Institute, Trinity College Dublin, D02 R590 Dublin, Ireland; 4 Dubowitz Neuromuscular Centre, UCL Great Ormond Street Institute of Child Health, 30 Guildford Street, London WC1N 1EH, UK; 5 SQY Therapeutics-Synthena, UVSQ, 78180 Montigny le Bretonneux, France

**Keywords:** ASO-based therapy, central nervous system, intracerebroventricular injection, intrathecal injection, antisense oligonucleotides, exon-skipping, Duchenne muscular dystrophy, CNS delivery

## Abstract

Nucleic acid-based therapies have demonstrated great potential for the treatment of monogenetic diseases, including neurologic disorders. To date, regulatory approval has been received for a dozen antisense oligonucleotides (ASOs); however, these chemistries cannot readily cross the blood–brain barrier when administered systemically. Therefore, an investigation of their potential effects within the central nervous system (CNS) requires local delivery. Here, we studied the brain distribution and exon-skipping efficacy of two ASO chemistries, PMO and tcDNA, when delivered to the cerebrospinal fluid (CSF) of mice carrying a deletion in exon 52 of the dystrophin gene, a model of Duchenne muscular dystrophy (DMD). Following intracerebroventricular (ICV) delivery (unilateral, bilateral, bolus vs. slow rate, repeated via cannula or very slow via osmotic pumps), ASO levels were quantified across brain regions and exon 51 skipping was evaluated, revealing that tcDNA treatment invariably generates comparable or more skipping relative to that with PMO, even when the PMO was administered at higher doses. We also performed intra-cisterna magna (ICM) delivery as an alternative route for CSF delivery and found a biased distribution of the ASOs towards posterior brain regions, including the cerebellum, hindbrain, and the cervical part of the spinal cord. Finally, we combined both ICV and ICM injection methods to assess the potential of an additive effect of this methodology in inducing efficient exon skipping across different brain regions. Our results provide useful insights into the local delivery and associated efficacy of ASOs in the CNS in mouse models of DMD. These findings pave the way for further ASO-based therapy application to the CNS for neurological disease.

## 1. Introduction

Antisense oligonucleotides (ASOs) hold tremendous therapeutic potential for many genetic diseases. ASOs are short, synthetic, single-stranded oligonucleotides that can bind to mRNA and non-coding RNAs to reduce, restore, or modify protein expression [1,2,3]. Several ASOs have already reached market approval, in particular for the treatment of neuromuscular disorders, such as spinal muscular atrophy (SMA) and Duchenne muscular dystrophy (DMD). DMD is a neuromuscular disease with an incidence of 1:5000 boys, who carry mutations in the *DMD* gene that disrupt the open-reading frame of the dystrophin protein (Dp) in muscle, heart, and brain [4,5]. The ASOs used in DMD aim to restore the open reading frame to produce an internally deleted but still functional protein [6,7,8]. Over the past decade, a number of ASOs have been developed to target exons flanking different relatively common groups of DMD mutations, leading to four such therapies being conditionally approved by the FDA. These ASOs require regular (weekly) systemic administration via an intravenous route. However, none of these ASOs can address DMD brain comorbidities, which have a significant impact on the patients’ quality of life. These include intellectual disability and neurobehavioral comorbidities, affecting between 30 and 50% of individuals with DMD [9,10]. The European *Brain Involvement in Dystrophinopathies* (BIND) consortium (https://bindproject.eu/,accessed on 6 February 2023) aims to investigate and characterize further the role of the various dystrophin isoforms in the CNS and evaluate the potential reversibility of the central deficits associated with the lack of dystrophin. In this context, we are using the exon 52-deleted *mdx52* mouse model of DMD [11], as this mutation is located in a “hot spot” region that is frequently mutated in DMD patients [10,12,13]. We previously showed that *mdx52* mice, lacking Dp427, Dp260, and Dp140, display enhanced anxiety and fearfulness and impaired associative fear learning as compared to the original Dp427 deficient-*mdx* mouse model [14]. The development of therapeutic approaches in this mouse model is thus of great interest, as it directly translates to patients’ conditions. As part of our investigations, we first aimed to optimize the administration route to achieve high and widespread delivery of ASOs in the CNS of DMD mouse models.

Considering that the systemic delivery of ASO requires high doses and results in very low efficacy in the CNS, mostly due to the inability of oligonucleotides to efficiently cross the blood–brain barrier (BBB), we focused on local delivery to the CNS. One of the most common delivery method to the CNS is administration to the cerebrospinal fluid (CSF) [15,16]. CSF continuously flows in cerebral ventricles, the subarachnoid, cisternal spaces, and the spinal canal. Its direct contact with the CNS makes it an ideal delivery route to achieve widespread distribution to the CNS [17], although different regions are targeted with different efficiencies. CSF delivery can be achieved via injection into the cerebral ventricles, the lumbar intrathecal space, or the *cisterna magna*. The most commonly used delivery route to the CSF, in particular for rodents, is via intracerebroventricular (ICV) injection based on specific stereotaxic coordinates [18]. On the other hand, intrathecal (IT) delivery via lumbar puncture is a convenient delivery strategy not only because it does not require a stereotaxic set up but also because of ease of access and minimal invasiveness [19,20,21]. This method results in efficient drug distribution to the spinal cord in animal models and patients and is successfully used in the clinic for the administration of the ASO Nusinersen to SMA patients [22]. However, preclinical data in animal models also suggest that lumbar intrathecal delivery results in limited distribution to the supratentorial brain structures [23], which are the target for DMD therapies. As an alternative to lumbar delivery, administration into the *cisterna magna* has been used in animal models to achieve delivery closer to brain structures. This method has resulted in widespread distribution to the cerebellum, hindbrain, and spinal cord in mice [23].

In the present study, we aimed to optimize ASO efficacy in the CNS, to reach the highest possible exon-skipping levels in brain regions known to express dystrophin, such as the cerebellum, hippocampus, and cortex. We therefore evaluated different delivery methods, in particular ICV and ICM (intra-cisterna magna), using various injection regimens. Notably, we compared unilateral vs. bilateral ICV injections and a bolus vs. slow rate of injection, as well as the repeated administration of ASO using a cannula and continuous administration using osmotic pumps. Finally, we studied the feasibility and resulting effects of combining both ICM and ICV delivery methods.

Considering that ASO biodistribution in the CNS may be affected by various factors, such as ASO chemistry and charge, we used two different chemistries of ASO in this study: the Phosphorodiamidate Morpholino Oligomer (PMO) chemistry and the tricyclo-DNA (tcDNA) chemistry. PMOs have a neutral charge and are already approved by the FDA for the systemic treatment of DMD [8], while tcDNA is a charged, lipid-conjugated ASO that has previously shown therapeutic potential in mouse models of DMD [24,25]. Overall, our detailed comparative study provides useful insights into the local delivery and associated efficacy of ASOs in the CNS of mouse models of DMD.

## 2. Materials and Methods

### 2.1. Animals and Antisense Oligonucleotides

*Mdx52* mice (B6;129S-*Dmd^tm1Mok^*) breeders were generously provided by Prof. Sasaoka Toshikuni and Dr. Motoya Katsuki (Department of Comparative & Experimental Medicine, Brain Research Institute, Niigata University, Japan) and kindly shipped to our laboratories by Dr. Jun Tanihata and Dr. Shin’ichi Takeda (National Center of Neurology and Psychiatry, Tokyo, Japan). *Mdx52* mice contain a neomycin cassette in place of exon 52 of the *DMD* gene on the X chromosome, thereby disrupting the *dmd* gene reading frame and eliminating the expression of Dp427, Dp260, and Dp140 dystrophin isoforms, but preserving the expression of Dp116 (in peripheral nerves) and of Dp71 (in brain and retina) [11] (for a review of DMD isoforms see [26]). Optimization experiments were performed using hDMD mice expressing the human dystrophin gene [27,28]. The mouse lines were backcrossed with the C57BL/6J strain for more than eight generations. At the animal facility Plateforme 2Care, UFR des Sciences de la santé, Université de Versailles-Saint Quentin (France), heterozygous females were crossed to C57BL/6JRj male mice to generate *mdx52* and littermate control (WT) males.

At Trinity Biomedical Sciences Institute (TBSI), Trinity College Dublin, heterozygous females were crossed to C57BL/6J males to generate *mdx52* and littermate control (WT) males. Genotypes were determined via PCR analysis of DNA from tail or ear biopsies. Mice were housed in individually ventilated cages (IVC, Tecniplast) in a specific pathogen-free facility on a 12 h light/dark cycle with access to food and water *ad libitum*. Animal care and all experimental procedures complied with the national and European legislation, approved by the French government (Ministère de l’Enseignement Supérieur et de la Recherche, Autorisation APAFiS #6518) and the Irish Health Products Regulatory Authority (Ref: AE19136/P131) and with the approval of the TCD Animal Research Ethics Committee.

The tcDNA-ex51 used in this study targets an exonic splicing enhancer within exon 51 of the dystrophin pre-mRNA (position +48 + 62, sequence GGAGATGGCAGTTTC) and was synthesized by SQY Therapeutics (Montigny-le-Bretonneux, France). Palmitic acid was conjugated at the 5′ end of the tcDNA full phosphodiester via a C6-amino linker and a phosphorothioate bond as previously described [24]. M51D PMO used in this study targets the *Dmd* exon 51 splice donor site (position + 10 − 15, sequence TTGTTTTATCCATACCTTCTGTTTG) (Genetools LLC, Philomath, QR, USA). For analysis of in situ biodistribution, a carboxyfluorescein-conjugated control PMO (CCTCTTACCTCAGTTACAATTTATA, Genetools LLC, Philomath, QR, USA) was used. PMO was resuspended in sterile phosphate buffered saline and heated to 65 °C for 15 min before use to ensure solubility.

ASO administrations were performed using 6–8-week-old *mdx52* and WT mice anesthetized via intraperitoneal injection of a ketamine (95 mg/kg)/medetomidine (1 mg/kg) mixture. For intracerebroventricular injections (ICV), tcDNA-Ex51 or M51D PMO solutions were bilaterally injected into the lateral brain ventricles (−0.5 mm anterior and 1 mm lateral from bregma; −2 mm from pia). A volume of 5 μL was infused into each ventricle, i.e., a total volume of 10 µL of ASO corresponding to 400 µg of tcDNA (71 nmol) and 900 µg for PMO (107 nmol). The slow-bolus rate of 0.3 μL/min was compared to a bolus rate of 0.6 µL/s, which was the fastest rate achievable with the injection system (Legato 130 pump from KD scientific). For intra-cisterna magna injections (ICM), 10 µL of tcDNA-Ex51 (i.e., 400 µg) or M51D PMO (i.e., 900 µg) solutions were administered, using a 30-gauge stainless steel needle with a point 4 style bevel curved (45°) 2 mm from the tip, so that it was J-shaped.

Repeated ICV delivery was performed using cannula implants in the lateral ventricles (−0.5 mm from bregma; 1 mm lateral; −2 mm from pia) and an infusion rate of 0.6 μL/min during each infusion, while the animals were under isoflurane anesthesia. Infusions were repeated between 3 and 5 times with a minimal recovery of 3 days between each infusion. For tcDNA, 3 and 5 infusions were performed corresponding to a total injected dose of 1 and 2 mg respectively (~176 and 352 nmol). For PMO, 4 infusions were performed corresponding to a total injected dose of 2.7 mg (~320 nmol).

For continuous slow delivery, osmotic pumps (Alzet, Model 1002) were implanted under the back skin and directly linked to the ventricle via a cannula. The osmotic pumps delivered a total volume of 100 µL of ASO solution (0.25 µL/h for 2 weeks), corresponding to up to 4 mg of tcDNA (~700 nmol) over the period of 2 weeks.

### 2.2. Biodistribution Analysis in Tissue Lysates

For tcDNA administrations, tissues were homogenized using the Precellys 24 (Bertin Instruments, France) in lysis buffer (100 mmol/L Tris–HCl, pH 8.5, 200 mmol/L NaCl, 5 mmol/L EDTA, 0.2% sodium dodecyl sulfate) containing 2 mg/mL of proteinase K (Invitrogen) (50 mg tissue/mL of buffer), followed by incubation overnight at 55 °C in a hybridization oven. After centrifugation at 14,000× *g* rpm (Sorval ST 8R centrifuge, 75,005,719× *g* rotor) for 15 min, tcDNA was quantified in the supernatant via a hybridization assay with a molecular beacon probe, as previously described [24]. Briefly, 10 µL of tissue lysates was incubated with a 5′ Cy3-DNA complementary probe conjugated with HBQ quencher at 3′ in a black non-binding 96-well plate (ThermoFischer Scientific, Rockford, IL, USA), PBS was added to a final volume of 100 µL per well, and fluorescence was measured on a spectrophotometer (Ex 544 nm/Em 590 nm using FluoStar Omega, BMG Labtech, France). The amount of tcDNA in tissues was determined using a standard curve built on the measurement of known tcDNA quantities dissolved in the respective tissue lysates of saline-treated animals.

### 2.3. Biodistribution Analysis In Situ

In situ analysis of the biodistribution of tcDNA was performed using sagittal and coronal brain cryosections (30 µm) from brains that were fresh-frozen in powdered dry ice. Cryosections were post-fixed in a 5 min bath of acetone/methanol (1:1), and detection of tcDNA-ASO was achieved using a complementary probe conjugated to biotin, which was then revealed with a Streptavidin Alexa Fluor™ 555 conjugate (ThermoFisher Scientific, USA). Images were taken at equivalent locations and exposure times using a laser scanning confocal microscope (Zeiss LSM 700 × 40 objective). Stacks of 9 to 12 images (1024 × 1024 pixels) spaced by 1 μm were recorded at a resolution of 156 nm/pixel. Scan tiles images (1024 × 1024 pixels) were taken using the ScanR Olympus HCS microscope, X40 objective.

For PMO biodistribution a carboxyfluorescien-conjugated PMO was administered via ICV or ICM. Forty-eight hours later, mice were transcardially perfused with PBS followed by 4% paraformaldehyde (PFA), and brains were harvested. Whole brains were post-fixed for 6 h in 4% PFA, followed by sucrose cryoprotection in increasing concentrations of sucrose in 1X PBS (15%, 30%) at 4 °C. A final incubation in 30% sucrose/30% OCT was performed before brains were embedded in OCT using methylbutane cooled with liquid nitrogen and stored at −80 °C.

Brains were cryosectioned sagittally and coronally (10 μm), stained with a nuclear stain, and mounted with Prolon Diamond antifade mountant (Invitrogen, Carlsbad, CA, USA). Whole sections were imaged at equivalent locations and exposure times using a digital slide scanner (NanoZoomer S60, Hamamatsu, Shizuaka, Japan, 40× objective) at a resolution of 220 nm/pixel.

### 2.4. Exon 51 Skipping Analysis

For PMO-injected mice, brain regions were microdissected and stored in RNAlater (ThermoFisher Scientific, Carlsbad, CA, USA) at 4 °C overnight to preserve the RNA, before removal of the RNAlater for storage at −80 °C. Total RNA was isolated from dissected brain structures using TRIzol reagent (ThermoFisher Scientific, Carlsbad, CA, USA) or an RNeasy mini kit (Qiagen, Hilden, Germany) according to the manufacturer’s instructions. For visualization of the exon-skipping efficacy on gels, aliquots of 1 µg of total RNA were used for RT-PCR analysis using the Access RT-PCR System (Promega, Madison, WI, USA) in a 50 μL reaction using the external primers Ex49F (5′-AAACTGAAATAGCAGTTCAAGC-3′) and Ex53R (5′-ACCTGTTCGGCTTCTTCCTT-3′). The cDNA synthesis was carried out at 55 °C for 10 min, followed by the PCR of 30 cycles of 95 °C (30 s), 58 °C (1 min), and 72 °C (1 min). PCR products were electrophoresed on 1.5% agarose gels.

Exon 51 skipping was also measured via Taqman quantitative PCR, as previously described (Aupy et al., 2020), using Taqman assays designed against the exon 50–51 junction (assay Mm.PT.58.41685801: forward: 5′-CAAAGCAGCCTGACCGT-3′; reverse: 5′-TGACAGTTTCCTTAGTAACCACAG-3′; probe: 5′- TGGACTGAGCACTACTGGAGCCT-3′) and exon 50–53 junction (forward: 5′-GCACTACTGGAGCCTTTGAA-3′; reverse: 5′-CTTCCAGCCATTGTGTTGAATC-3′; probe: 5′-ACAGCTGCAGAACAGGAGACAACA-3′) (Integrated DNA technology). One hundred and fifty nanograms of cDNA was used per reaction, and assays were carried out in triplicate. Assays were performed under fast cycling conditions on a Biorad CFX384 Touch Real-Time PCR Detection System, and data were analyzed using the absolute copy number method. For a given sample, the copy number of skipped products (exon 50–53 assay) and unskipped products (exon 50–51 assay) were determined using the standards Ex49-54Delta52 and Ex49-54Delta51+52, respectively (gBlocks gene fragments from Integrated DNA technology). Exon 51 skipping was then expressed as a percentage of total dystrophin (calculated by the addition of exon 50–51 and exon 50–53 copy numbers).

### 2.5. Statistical Analysis

Data are presented as means ± SEMs; statistics were performed using the GraphPad Prism8 software (San Diego, CA, USA). Repeated measures were analyzed using repeated-measure (RM) two-way analysis of variance (ANOVA) with treatment as the between-group factor and brain structure as the within-subjects factor. Significant levels were set at * *p* < 0.05, ** *p* < 0.01, *** *p* < 0.001, and **** *p* < 0.0001.

## 3. Results

### 3.1. Single ICV ASO Delivery Optimization

We first focused on one of the most commonly utilized delivery routes to the CNS in rodents, intracerebroventricular (ICV) administration (Figure S1), and evaluated the different parameters for ASO delivery. Considering that the injection volume to the ventricles is restricted to a maximum of 5 µL/hemisphere and that ASO chemistries display limited solubility, we first compared bilateral ICV injection with unilateral injection.

**Figure 1:**
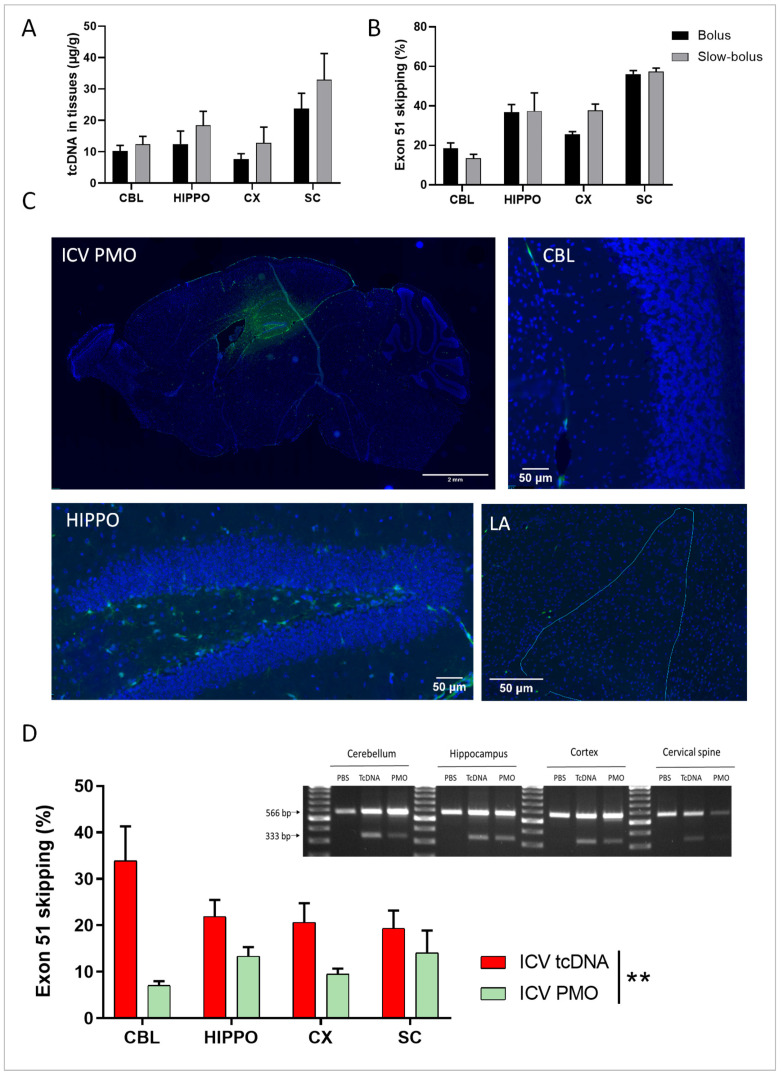
Quantification of tcDNA and PMO in specific brain regions and exon skipping after single ICV delivery. (**A**) Quantification by fluorescent hybridization assay of tcDNA-Ex51 content in various CNS tissues 4 weeks after the ICV administration using a bolus or a slow-bolus rate. Results are expressed as means ± SEMs; n = 4 hDMD-tcDNA-Ex51. (**B**) Quantification of exon 51-skipping levels via RT-qPCR in different brain tissues 4 weeks after the ICV administration using a bolus or a slow-bolus rate. Results are expressed as means ± SEMs; n = 4 hDMD-tcDNA-Ex51. (**C**) Carboxyfluorescein-conjugated PMO detected 48 h post-ICV slow-bolus rate delivery. Representative images show a sagittal section (scale bar 2 mm), cerebellum lobules IV-V (CBL), hippocampus dentate gyrus (HIPPO), and the lateral amygdala (LA), n = 3, (scale bars 50 µm). (**D**) Left panel: quantification of exon 51-skipping levels via RT-qPCR in different brain tissues after a slow-bolus rate ICV administration. Right panel: detection of exon 51-skipped dystrophin mRNA via RT-PCR in different brain tissues 7 weeks after the ICV administration of saline (PBS), tcDNA-ex51 (n = 5), or PMO-ex51 (n = 3) in mdx52 mice. CBL: cerebellum, CX: cortex, HIPPO: hippocampus, and SC: spinal cord (cervical region). ** *p* < 0.01 between tcDNA and PMO treatment analyzed via RM two-way ANOVA.

Adult hDMD mice received either a unilateral ICV injection of 200 µg of tcDNA-ex51 (previously described in [25]) or a bilateral ICV injection of 2 × 200 µg (i.e., a total amount of 400 µg of ASO). We first checked that unilateral ICV injection results of the distribution of ASO to both hemispheres of the brain (Figure 1A) (hemisphere effect *p* = 0.5596). While it could be expected that bilateral injection of 400 µg of ASO would lead to increased ASO levels in tissue relative to that with unilateral ICV injection of 200 µg, the concentrations in tissues were surprisingly much higher than the expected 2-fold difference following the bilateral delivery (~7.7-fold on average) (Figure 1A–C) (*p* = 0.0031 unilateral vs. bilateral). Likewise, exon 51-skipping efficacy was measured via quantitative RT-PCR and revealed that bilateral ICV injection induced significantly higher levels of exon skipping (~6-fold change) compared to that with unilateral ICV injection (*p* < 0.0001 unilateral vs. bilateral) (Figure 1D). Based on these results, we selected bilateral injection as our standard ICV injection method for further experiments.

Next, as different rates of administration have been described in the literature [25,29], we chose to examine rapid-bolus delivery (0.6 µL/s) versus a slow-bolus rate (0.3 µL/min) for the administration of tcDNA-ASO in adult hDMD mice [28]. Both methods led to a homogenous distribution of tcDNA-Ex51 to the different CNS regions (rate effect *p* = 0.1783), although slightly higher levels of ASO were detected in the slow-bolus rate-injected tissues (Figure 1A). Exon 51-skipping levels were similar for both rates (rate effect *p* = 0.51) (Figure 1B). Considering that 0.3 µL/min is closer to the cerebrospinal fluid (CSF) flow [30] we selected this slow-bolus rate to compare PMO and tcDNA delivery in *mdx52* mice. Eight-week-old *mdx52* mice received a bilateral ICV injection of a carboxyfluorescein-conjugated PMO in order to investigate its biodistribution. Forty-eight hours after the injection, PMO was mainly detected in the hippocampus, with some in the cortex and only very limited diffusion to the cerebellum and amygdala (Figure 1C and Figure S2A). For tcDNA, we performed in situ hybridization using a complementary probe to directly detect the injected tcDNA-ex51, thus avoiding the need to inject a fluorescent ASO. While we were not able to easily detect the tcDNA 48 h after the ICV injection using this technique, we could clearly detect it 3 weeks and 7 weeks post-ICV (Supplementary Figure S2B,C). The tcDNA-ASO was found at a high concentration in the CA1 region of the hippocampus and in the cerebellum at both time points.

The efficacy of both PMO and tcDNA-ASOs targeting the *Dmd* exon 51, administered via bilateral ICV injection, were next compared in 8-week-old *mdx52* mice. The highest feasible dose was injected in both cases (400 µg for tcDNA and 900 µg for PMO, corresponding to 72 and 107 nmol, respectively). Seven weeks post-ICV, the levels of exon 51 skipping were measured via qPCR and showed that both tcDNA and PMO induced homogenous skipping across the different CNS structures (structure effect *p* = 0.193). The tcDNA, however, showed significantly higher efficacy in inducing exon 51 skipping, with a mean of 20–30%, compared to that with PMO, which induced 7–15% of exon 51 skipping (ASO chemistry effect *p* = 0.0013) (Figure 1D). The difference was particularly striking in the cerebellum. Exon 51 skipping in the different brain regions was also visualized on the gel after RT-PCR for both chemistries (Figure 1D right panel).

### 3.2. Repeated ICV ASO Delivery

**Figure 2:**
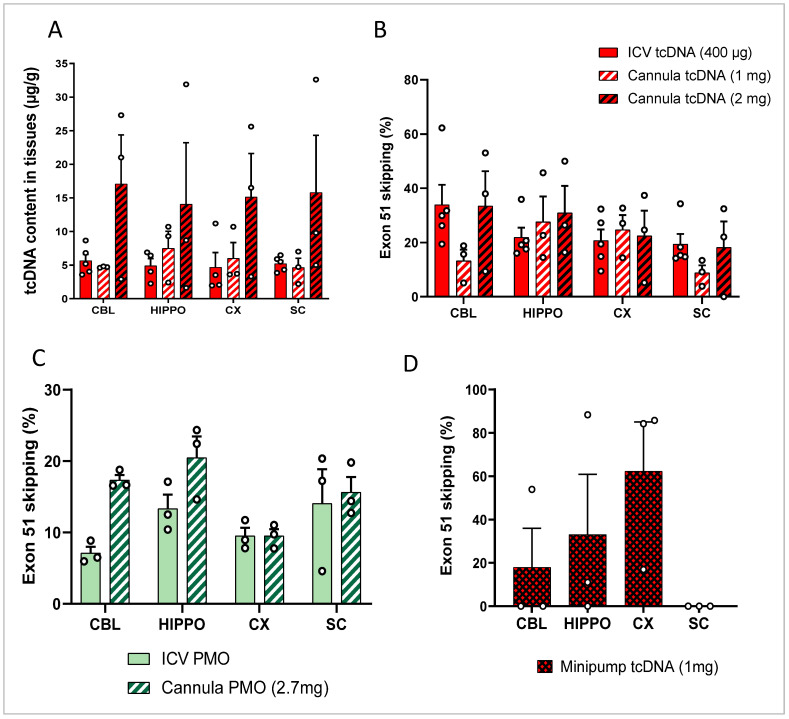
Repeated ICV delivery of tcDNA and PMO. (**A**) Quantification by fluorescent hybridization assay of tcDNA-Ex51 content in various CNS tissues after three multiple injections via a cannula implant 4 weeks after tcDNA administration. Results are expressed as means ± SEMs; n = 3 *mdx52*-tcDNA-Ex51 in cannula groups. (**B**) Quantification of exon 51-skipping levels via RT-qPCR in different brain tissues. Results are expressed as means ± SEMs; n = 3 *mdx52*-tcDNA-Ex51 in cannula groups. (**C**) Quantification of exon 51-skipping levels via RT-qPCR in different brain tissues 7 weeks after either single ICV slow-bolus injection or three administrations via a cannula implant of PMO. (**D**) Quantification of exon 51-skipping levels via RT-qPCR in different brain tissues after very slow diffusion via osmotic pumps of tcDNA. CBL: cerebellum, CX: cortex, HIPPO: hippocampus, and SC: spinal cord (cervical region). Results are expressed as means ± SEMs; n = 3 *mdx52*-minipump-tcDNA-Ex51.

In order to investigate whether it was possible to increase the levels of exon-skipping further, we evaluated the delivery of higher doses of ASOs. For this purpose and accounting for the maximum feasible dose for single injections, we performed repeated administrations of ASOs. Given that repeated ICV injection presents a high risk of damage to the cortex, cannula implants are commonly used for this purpose. Cannulas were implanted directly into the lateral ventricles, and delivery was repeated using anesthetized 8-week-old *mdx52* mice over a period of 2 weeks with at least 3 days between the administrations to allow for recovery. For tcDNA, two distinct treatment groups were analyzed, one receiving three injections of tcDNA (total amount, 1 mg; i.e., 176 nmol) and another group receiving five injections (total amount, 2 mg; i.e., 352 nmol). Mice treated with 2 mg of tcDNA showed tolerability issues characterized by a progressive reduction in mobility and a lack of responsiveness with weight loss. This group was thus euthanized 4 weeks after the last administration due to tolerability issues. The tcDNA quantification revealed homogeneous biodistribution through the CNS for all doses examined, although the 2 mg cannula group showed large inter-individual variability (Figure 2A) (structure effect *p* = 0.8533). Exon-skipping levels were also homogeneous in the different structures analyzed for all groups (Figure 2B). A large variability in exon-skipping levels was also observed in the 2 mg cannula group, and despite a slight tendency of this group to show higher skipping efficacy, we found no significant difference when compared to the 1 mg group or to a single ICV of 400 µg (dose effect *p* = 0.5603). These results were confirmed in the hDMD mouse model, in which we did not detect significantly higher exon skipping in the 2 mg cannula group compared to that with single ICV delivery (Figure S3A).

The repeated ICV delivery of PMO using a cannula (total amount, 2.7 mg; i.e., 320 nmol) was well tolerated and induced slightly higher exon 51-skipping levels in the various brain regions compared to that with the single bilateral ICV injection, although this was not statistically significant (*p* = 0.09 between ICV of 900 µg and repeated ICV of 2.7 mg, analyzed via RM two-way ANOVA) (Figure 2C).

### 3.3. ICV Delivery of ASO Using Osmotic Pumps

To investigate further the possibility of achieving greater efficacy levels, we evaluated the administration of even higher doses of ASO. For that purpose, we used osmotic pumps to deliver the ASO directly to the lateral ventricles at a very slow diffusion rate. This method allows for the delivery of larger volumes (up to 100 µL) of ASO over a sustained period of time (2 weeks), thus allowing for higher dosages of ASO reaching up to 4 mg of tcDNA-ASO (i.e., 704 nmol). Preliminary experiments were conducted in hDMD mice, which received either 2 or 4 mg of tcDNA-ex51. Mice were euthanized 4 weeks after the last administration because of tolerability issues (as reported above), and tissues were taken for analysis. Quantification of exon 51 skipping revealed lower levels in both osmotic pump groups compared to levels that were obtained after a single bilateral ICV injection of 400 µg (analyzed at 7 weeks) (Figure S3B). For experiments in *mdx52* mice, we lowered the dose to 1 mg to improve tolerability and this amount was indeed better tolerated. Mice were analyzed 7 weeks post-treatment, and exon-skipping levels were examined in the various brain regions (Figure 2D). This very slow diffusion method led to exon 51-skipping levels of around 18% in the cerebellum, 33% in the hippocampus, and 62% in the cortex (Figure 2D), but this was not statistically different from those with the bilateral single ICV injection of 400 µg (*p* = 0.7148 between the two treatments analyzed via two-way ANOVA), probably due to the very large variability between individuals in the osmotic pump group, making this delivery route less reliable.

### 3.4. Intra-Cisterna Magna Delivery of ASOs

**Figure 3:**
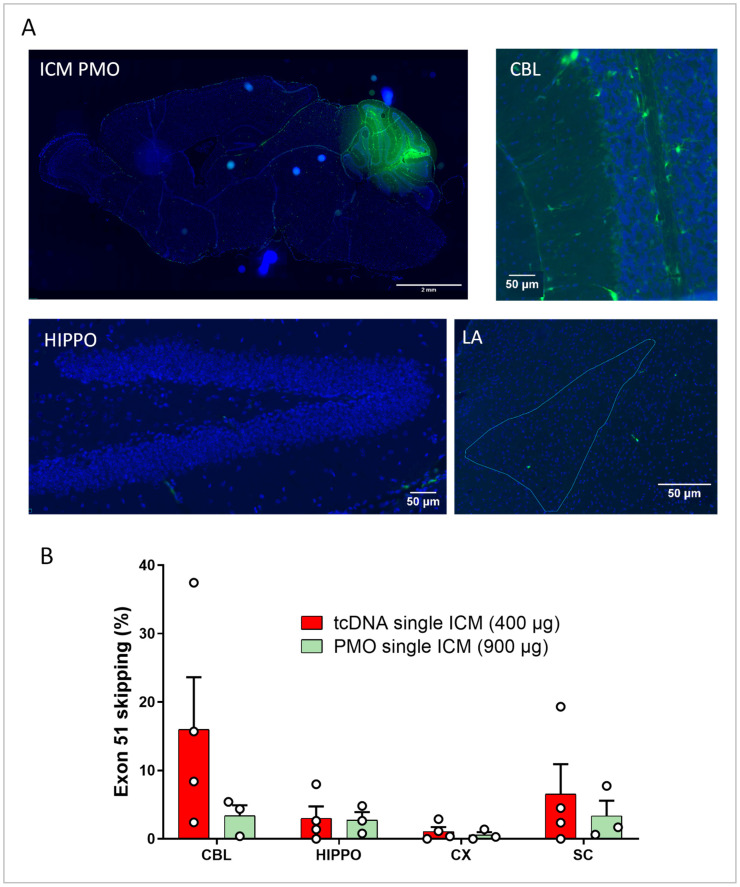
Single ICM delivery of tcDNA or PMO. (**A**) Carboxyfluorescein-conjugated PMO detected 48 h post-ICM delivery. Representative images showing a sagittal section (scale bar 2 mm), cerebellum lobules IV-V (CBL), hippocampus dentate gyrus (HIPPO), and the lateral amygdala (LA) (scale bars 50 µm); n = 3. (**B**) Quantification of exon 51-skipping levels via RT-qPCR in different brain regions (CBL: cerebellum, CX: cortex, HIPPO: hippocampus, and SC: spinal cord) after a single ICM administration of tcDNA or PMO. Results are expressed as means ± SEMs; n = 3 *mdx52*-PMO and n = 4 *mdx52*-tcDNA. RM two-way ANOVA: structure effect *p* = 0.1810; ASO chemistry effect *p* = 0.2623.

We next investigated other CNS delivery methods that may be better tolerated and more translatable for clinical use. In particular, we delivered ASO to the CSF through intra-cisterna magna (ICM) delivery. ICM is a less invasive method than ICV, allowing for delivery directly to the spinal canal or subarachnoid space without interference with any CNS tissue, making its repeated delivery possible. The ICM route of ASO administration tends to induce higher distribution to brain regions, such as the cerebellum, hindbrain, and hippocampus, while the intrathecal route via lumbar puncture (LP) more efficiently targets the spinal cord [23]. For these reasons, we focused on ICM delivery and validated this injection technique first using a blue dye (Figure S4A). Six-week-old *mdx52* mice received an ICM delivery of blue dye, and 5 min later, the mice were euthanized and the brains were harvested. The presence of the dye was observed throughout the ventricular system indicating the correct needle placement and angle at the base of the skull. As previously performed for the ICV studies, we then determined the biodistribution of ASO 48 h following the ICM injection of a carboxyfluorescein-conjugated PMO (Figure 3A and Figure S4). The PMO was detected brightly in the cerebellum, mostly near blood vessels, but only detected at very low levels in other brain regions. For the tcDNA, we performed fluorescent in situ hybridization 7 weeks post-ICM delivery, and high levels of ASO were also found in the cerebellum, while very little was found in the CA1 region of the hippocampus (Figure S4B).

We next assessed the molecular efficacy following single ICM injection of the maximum feasible dose of tcDNA and PMO targeting *Dmd* exon 51 (400 µg and 900 µg, respectively, as previously for the ICV injection). We examined exon-skipping levels and found a larger variability for the tcDNA as compared to that with the PMO (Figure 3B), which was further supported by an examination of the biodistribution of the tcDNA in brain structures (Figure S4C). The skipping quantification showed relatively low levels as compared to those with the ICV injection, 5 to 15% for the tcDNA depending on the structure and less than 5% in all analyzed structures for the PMO (ASO chemistry effect *p* = 0.2623) (Figure 3B).

### 3.5. Repeated ICM ASO Administration

**Figure 4:**
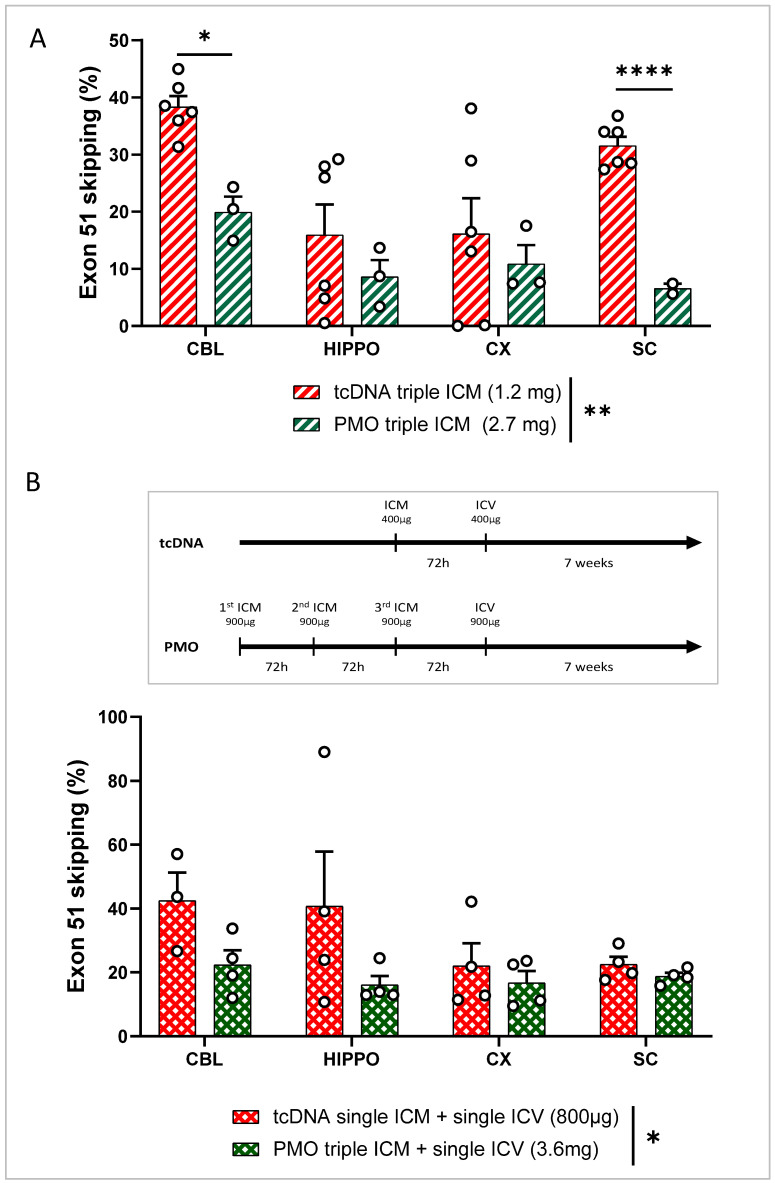
Repeated ICM delivery and combined ICM and ICV delivery of tcDNA or PMO in the CNS of *mdx52* mice. (**A**) Quantification of exon 51-skipping levels via RT-qPCR in different brain regions (CBL: cerebellum, CX: cortex, HIPPO: hippocampus, and SC: spinal cord) after PMO or tcDNA triple ICM 7 weeks after administration. Results are expressed as means ± SEMs; n = 6 *mdx52*-tcDNA and n = 3 *mdx52*-PMO. RM two-way ANOVA: structure effect *p* = 0.0182, ASO chemistry effect ** *p* = 0.0054. (**B**) Quantification of exon 51-skipping levels via RT-qPCR in different brain regions (CBL: cerebellum, CX: cortex, HIPPO: hippocampus, and SC: spinal cord) after PMO triple ICM + ICV injection and tcDNA single ICM + ICV injection, analyzed 7 weeks after administration. Results are expressed as means ± SEMs; n = 4 *mdx52*-tcDNA and n = 4 *mdx52*-PMO. RM two-way ANOVA: structure effect *p* = 0.3012, ASO chemistry effect * *p* = 0.0209. (* *p* < 0.05, **** *p* < 0.0001).

As the ICM delivery allows for less invasive repeated administration, we evaluated the effect of repeated ICM injections. PMO and tcDNA were delivered three times to 8-week-old *mdx52* mice at 72 h intervals, reaching total amounts of 2.7 mg for PMO (320 nmol) and 1.2 mg for tcDNA (213 nmol). The skipping efficacy showed that overall, the triple ICM injection led to significantly higher skipping levels than single ICM injection for both tcDNA (20–40%) and PMO (10–20%) (Figure 4A and Figure S4D,E). A comparison between tcDNA and PMO revealed higher levels of exon skipping induced by tcDNA (treatment effect *p* = 0.0054), as previously shown for ICV injections; however, high doses of PMO were better tolerated by the mice than high doses of tcDNA.

Finally, given that triple ICM injection still did not induce higher exon-skipping levels than our standard bilateral ICV injection, we tried to combine ICM and ICV delivery to increase both the efficacy and the distribution of ASO in the CNS. For PMO, *mdx52* mice received three ICM injections followed by an ICV injection as shown in Figure 4B, reaching a total amount of 3.6 mg of PMO. For tcDNA, considering that amounts totaling 1 mg or more were not well tolerated by the mice, *mdx52* mice received a single ICM injection before the ICV administration, reaching a total of 800 µg of tcDNA, which was well tolerated. Seven weeks after the ICV injection, exon-skipping levels were quantified and revealed an overall higher efficacy for the tcDNA-treated group (ASO chemistry effect *p* = 0.0209) compared to that in the PMO-treated group. However, these levels were not significantly higher than those obtained after the bilateral ICV injection of 400 µg of tcDNA (*p* = 0.73 between the two treatments analyzed via two-way ANOVA) (Figure S6A). Reassuringly however, for PMO, this combined delivery method was the most efficient one, resulting in the highest levels of exon 51 skipping obtained with this chemistry (16–22%) (Figure S6B,C).

## 4. Discussion

In this study, we have used the delivery of ASO therapies directly to the CNS as a means to bypass the BBB, in DMD neuromuscular mouse models. We compared two different oligonucleotide chemistries: the charge-neutral PMO chemistry, which is already approved by the FDA for the treatment of DMD following intravenous delivery, and the charged, lipid-conjugated tcDNA. TcDNA is an alternative chemistry that previously showed higher efficacy in mouse models of DMD and will enter clinical evaluation in H1 2023 (clinical trial *Avance 1*, sponsored by SQY Therapeutics). We focused on delivery to the CSF as opposed to direct injections into specific structures (e.g., intra hippocampal, etc.) in order to allow a broad distribution of ASO throughout the whole CNS since dystrophin isoforms are expressed across most CNS structures [31]. We therefore gathered useful information for the potential future use of ASOs to address dystrophin deficiency in the brain.

In the first part of the study, we focused on ICV administration, which has been commonly used to deliver therapeutic agents to the CNS in rodents; however various injection rates have been described in the literature. Rigo and colleagues previously demonstrated an increased ASO potency using a rapid-bolus rate (1 µL/s) of injection compared to that with a very slow rate of 0.5 µL/h [29]. Here, we compared a bolus rate of 0.6 µL/sec (the maximum bolus rate achievable with our injection system) to a slow-bolus rate of 0.3 µL/min, closer to the CSF flow [30]. Our results showed no statistical difference in the resulting ASO content or exon-skipping efficacy between both rates. Considering that we successfully used the slow-bolus rate in previous studies [25] and that it is closer to CSF flow, we selected this rate for our standard ICV protocol. The bilateral ICV administration of the maximal feasible dose of tcDNA induced relatively homogenous exon-skipping efficacy across the various CNS regions, ranging from 20 to 34%. Exon-skipping levels were also homogenous within the various brain structures following PMO ICV delivery, although they were significantly lower (ranging from 7 to 14%) compared to those obtained with tcDNA.

We next used cannula implants in order to deliver higher doses of ASO delivered to the CSF. The repeated injection of PMO, reaching up to 2.7 mg, induced slightly higher exon-skipping levels than single bilateral ICV delivery. Yet the difference was not statistically significant and efficacy levels did not correlate with the higher dose administered (2.7 vs. 0.9 mg). For tcDNA, repeated delivery through a cannula induced similar levels of exon skipping compared to those with single bilateral ICV, despite the much higher doses injected. This may be explained by the type of anesthesia used during the delivery. In order to avoid repeated long anesthesia sessions, mice implanted with the cannula were subsequently anaesthetized under isoflurane when receiving the tcDNA, whereas mice from the ICV bilateral groups were anaesthetized using ketamine/medetomidine when receiving the ASO. Indeed, several studies have recently raised the impact of anesthesia on CSF flow and showed that distribution to the CSF may be different depending on the wakefulness state [32]. Moreover, Ma and colleagues demonstrated that the CSF efflux out of the brain is higher under isoflurane anesthesia compared to that under deeper chemical anesthesia using ketamine/medetomidine [33]. This suggests that ASO may be more quickly eliminated from the CSF under isoflurane anesthesia (i.e., during delivery via a cannula) than under deeper anesthesia (i.e., bilateral ICV). This hypothesis could also explain the results obtained with the osmotic pumps, which delivered the ASO to the CSF in awake mice, since the clearance of CSF-infused tracers was shown to be increased under conscious conditions [33]. These findings are also in line with those previously reported by Rigo and colleagues that demonstrated the lower efficacy of a very slow rate of infusion (0.5 µL/h), similar to the one of the osmotic pumps that deliver the ASO continuously through a catheter implanted in the lateral ventricle, at a slow rate of 0.25 µL/h for 2 weeks in conscious animals.

Besides the lack of improvement in exon-skipping efficacy, the administration of a high dose of tcDNA was not very well tolerated by the mice, which showed general reduced mobility and weight loss with hypoactivity and a lack of responsiveness. Such toxicities have previously been observed following the direct injection of charged ASO in the CNS of rodents [34,35,36] and highlight the need to perform detailed safety studies before considering the potential future application to DMD patients [37]. Furthermore, we detected particularly high variability with this procedure, thus questioning its reproducibility. PMO-treated mice did not show any tolerability issues even after receiving a very high dose up to 2.7 mg of PMO with the cannula. Yet, exon-skipping levels obtained after the repeated delivery of PMO were still lower than those obtained after a single bilateral ICV injection of tcDNA.

We next investigated ICM injection as an alternative delivery to the CSF. Because ICM injections are less invasive than ICV procedures, this route may offer an easier opportunity for repeated administration. In addition, IT delivery is well tolerated and already approved for ASO delivery in the clinic for SMA [38]. The biodistribution following ICM injection differs from that of ICV as it preferentially targets the cerebellum, hindbrain, olfactive bulb, and spinal cord in mice [23] and therefore offers an interesting alternative delivery route for ASOs. We indeed clearly detected both PMO and tcDNA in the cerebellum after a single ICM injection, while only limited signal was detected in the hippocampus and cortex. This translated into higher exon-skipping levels in the cerebellum and spinal cord of tcDNA-treated mice, which are the closest regions to the injection site. Single ICM injections induced lower efficacy than ICV for both PMO and tcDNA; however, triple ICM achieved significantly higher exon-skipping levels than single ones. Considering that the wakefulness state of the animal may have an impact on the CSF efflux and influence ASO biodistribution and efficacy as discussed above, we decided to perform the ICM administrations under ketamine/medetomidine anesthesia as opposed to isoflurane. Indeed, besides the previously mentioned studies investigating the impact of anesthesia on CSF distribution [32,33], Xavier and colleagues evaluated this parameter in the context of a cannula implant into the *cisterna magna* and recommended a mixture of ketamine/xylazine for chronic injection [39]. This may explain why repeated ICM delivery induced higher efficacy than single ICM injection, while this was not the case for repeated ICV compared to single ICV.

Finally, we investigated the combination of ICV and ICM delivery with the objective of reaching the highest achievable efficacy levels but also a wider CNS distribution of the ASO. Administration of up to 3.6 mg of PMO to the CSF was well tolerated by the mice and led to homogenous exon 51-skipping levels across the different brain structures ranging from 16% to 22%. This combined administration was the most efficient delivery method for PMO (Figures S6B and S7). In order to avoid tolerability issues observed with >1 mg of tcDNA, ICV injection of tcDNA was combined with a single ICM administration reaching 800 µg of tcDNA, which was well tolerated. This combined method also resulted in the highest efficacy levels for tcDNA ranging from 22 to 42% of exon-skipping levels, although not significantly different from those with the bilateral ICV administration of 400 µg (ranging from 19 to 34%).

## 5. Conclusions

Together, our study highlights the importance of the delivery route, as well as the impact of the ASO chemistry and the type of anesthesia used during injection, on ASO distribution and molecular efficacy in the CNS. Overall, these results provide useful insights into the direct delivery of ASOs to the CNS of mice and lay the foundations for future work investigating the effects of ASO therapy in the CNS in mouse models of DMD. This work, linked to an assessment of functional rescue of the neuro-behavioral abnormalities that have been described in dystrophic mice, paves the way for the potential future clinical application of CNS-delivered ASO for individuals affected by DMD.

## Data Availability

The primary data for this study are available from the authors upon request.

## Supplementary Materials

The following supporting information can be downloaded at: https://www.mdpi.com/article/10.3390/cells12060908/s1, Figure S1: Schematic representation of the various injection methods and anatomical localization of the brain regions dissected for molecular analysis; Figure S2: Comparison of unilateral of bilateral ICV injection of tcDNA-ASO in hDMD mice; Figure S3: In situ biodistribution of PMO and tcDNA following single ICV administration; Figure S4: Comparison of single and repeated ICV delivery of tcDNA in hDMD mice; Figure S5: Biodistribution of ASO following ICM delivery; Figure S6: Comparison of various delivery routes for tcDNA and PMO; Figure S7: Summary of exon skipping efficacy following various delivery routes for tcDNA and PMO.
