# Supplementary material for: Investigating the Impact of Delivery Routes for Exon Skipping Therapies in the CNS of DMD Mouse Models
Source: Cells. 2023 Mar 15;12(6):908. doi: 10.3390/cells12060908 (PMC10047648; doi:10.3390/cells12060908)
Supplement: Supplementary file 1 [file cells-12-00908-s001.zip › cells-2247414-supplementary.pdf]

Supplemental Information for the manuscript:

Investigating the impact of delivery routes for exon skipping therapies in the CNS of DMD mouse models

Amel Saoudi[1,2], Claire Fergus[3], Talia Gileadi[4], Federica Montanaro[4], Jennifer Morgan[4], Vincent P. Kelly[3], Thomas Tensorer[5], Luis Garcia[1], Cyrille Vaillend[2], Francesco Muntoni[4] and Aurélie Goyenvalle[1*]

Supplemental Information contains:

- Supplementary figure 1: Schematic representation of the various injection methods and anatomical localization of the brain regions dissected for molecular analysis
- Supplementary figure 2: Comparison of unilateral of bilateral ICV injection of tcDNA-ASO in hDMD mice.
- Supplementary figure 3: In situ biodistribution of PMO and tcDNA following single ICV administration.
- Supplementary figure 4: Comparison of single and repeated ICV delivery of tcDNA in hDMD mice.
- Supplementary figure 5: Biodistribution of ASO following ICM delivery.
- Supplementary figure 6: Comparison of various delivery routes for tcDNA and PMO.
- Supplementary figure 7: Summary of exon skipping efficacy following various delivery routes for tcDNA and PMO.

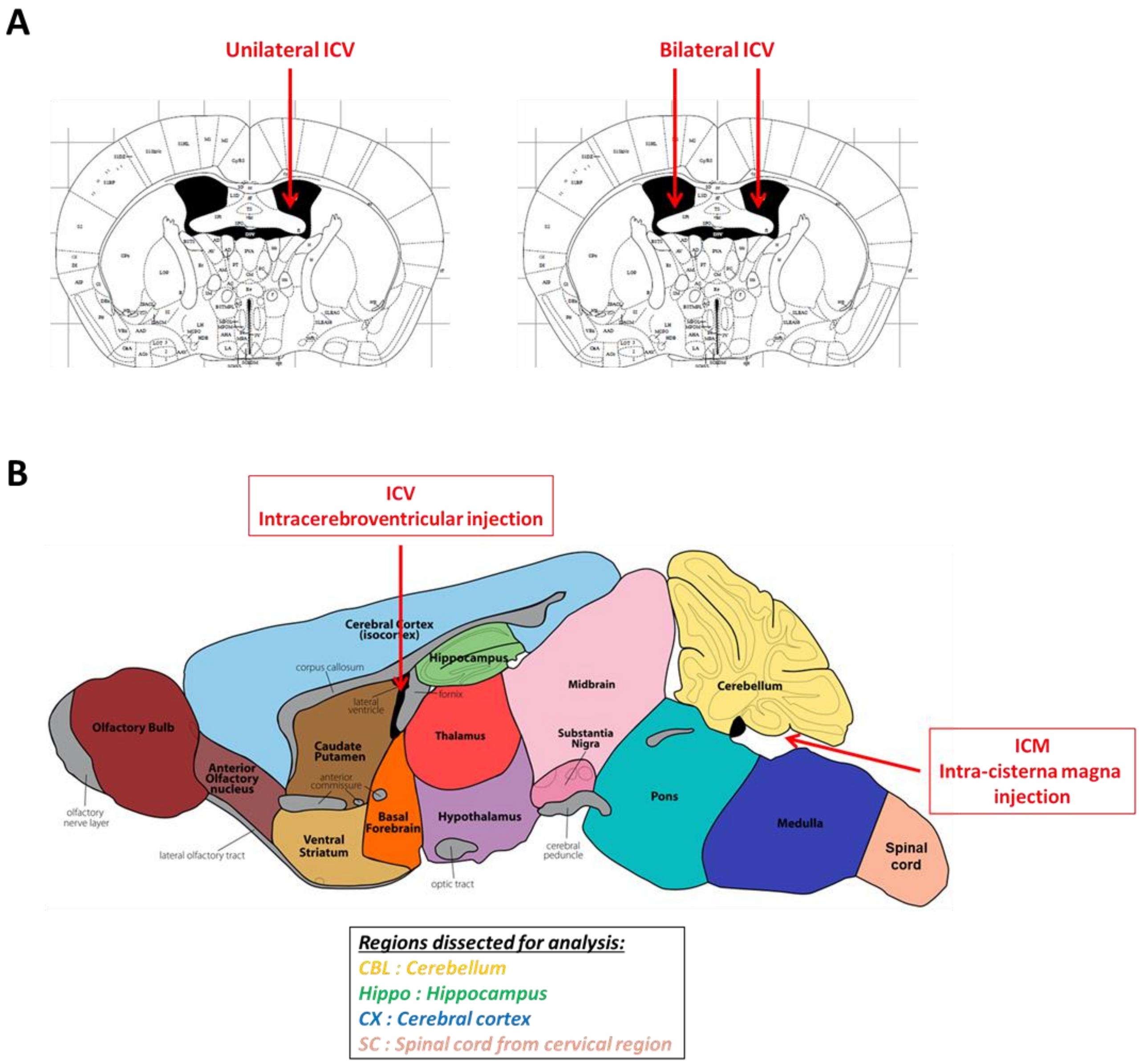

Supplementary figure 1: Schematic representation of the various injection methods and anatomical localization of the brain regions dissected for molecular analysis. A) representation of coronal sections and B) representation of a sagittal section.

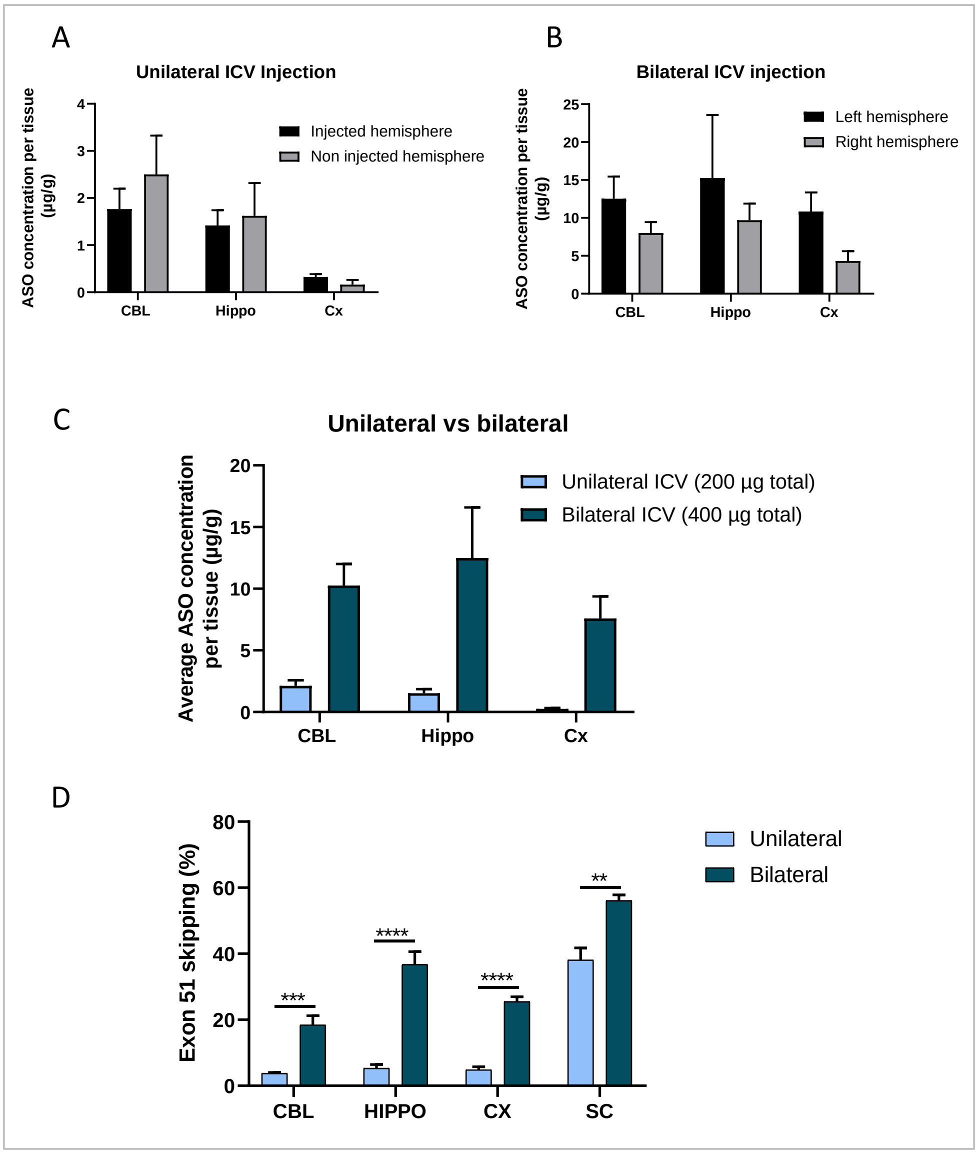

Supplementary figure 2: Comparison of unilateral of bilateral ICV injection of tcDNA-ASO in hDMD mice. (A) Quantification by fluorescent hybridization assay of tcDNA-Ex51 content in different CNS regions after a unilateral ICV injection of 200 µg of tcDNA-Ex51. Results are expressed as means ± SEM; n=4 hDMD-tcDNA-Ex51. (B) Quantification by fluorescent hybridization assay of tcDNA-Ex51 content in different CNS regions after a bilateral ICV injection of 400 µg of tcDNA-Ex51 (200 µg in each hemisphere). Results are expressed as means ± SEM; n=4 hDMD-tcDNA-Ex51. (C) Quantification by fluorescent hybridization assay of tcDNA-Ex51 content in different CNS regions after unilateral or bilateral ICV delivery of tcDNA (200 or 400 µg respectively). Results are expressed as means ± SEM; n=3 hDMD. (D) Quantification of exon 51-skipping levels by RT-qPCR in different brain regions after unilateral or bilateral ICV delivery of tcDNA (200 or 400 µg respectively). Results are expressed as means ± SEM; n=3 hDMD. CBL: cerebellum, CX: cortex, HIPPO: hippocampus and SC: spinal cord (cervical region). Statistical analysis: RM two-way ANOVA (**p<0.01, ***p<0.005, ****p<0.001).

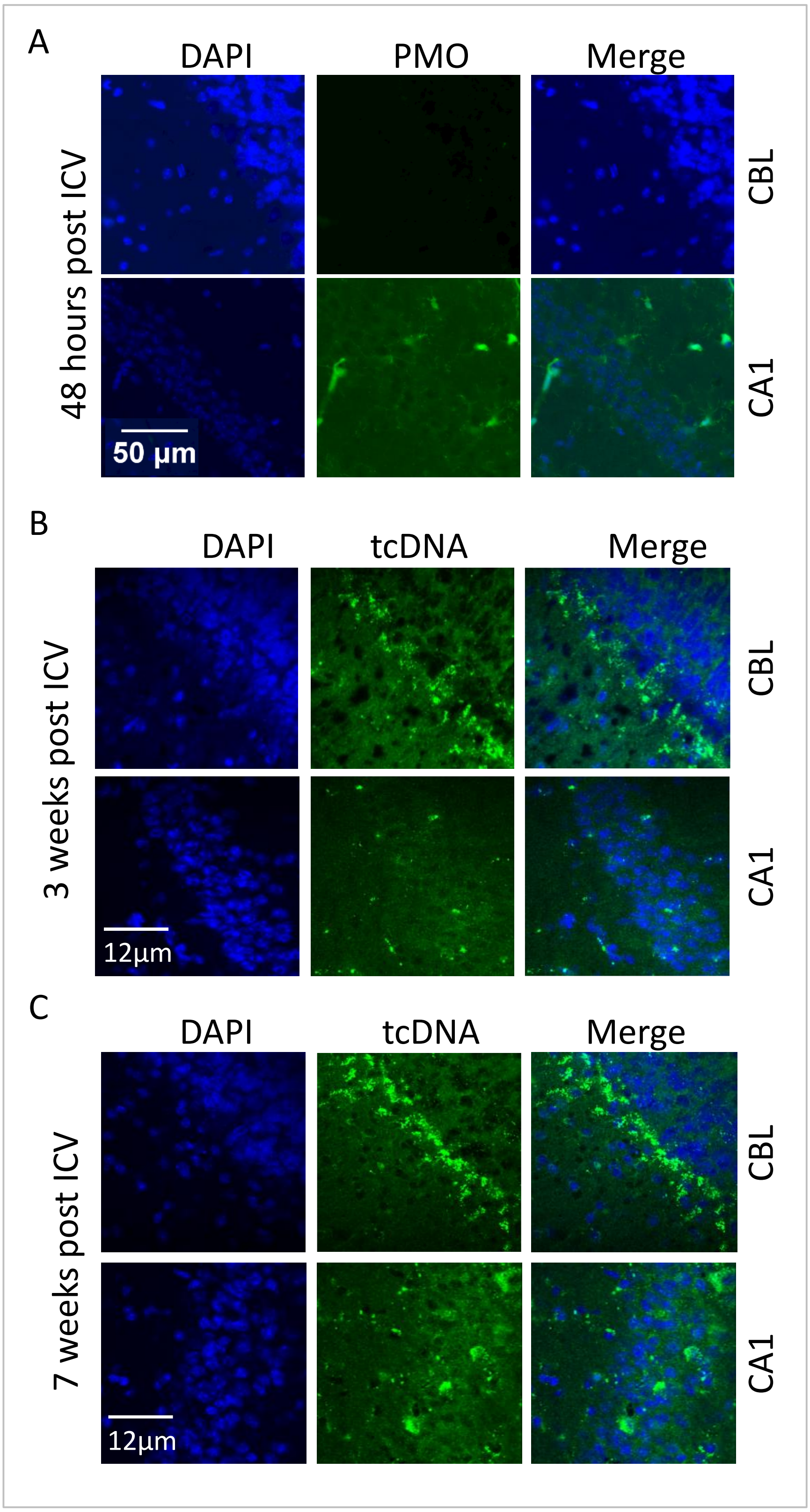

Supplementary figure 3: *In situ* biodistribution of PMO and tcDNA following single ICV administration. (A) Carboxyfluorescein-conjugated PMO detected 48 hours after ICV injection in the hippocampus (CA1) and cerebellum (CBL). Scale bar 50µm. (B) Fluorescent *in situ* hybridization to detect tcDNA-Ex51 3 weeks after ICV injection in hippocampus (CA1) and cerebellum (CBL). (C) Fluorescent *in situ* hybridization to detect tcDNA-Ex51 7 weeks after ICV injection in hippocampus (CA1) and cerebellum (CBL). Scale bar 12µm.

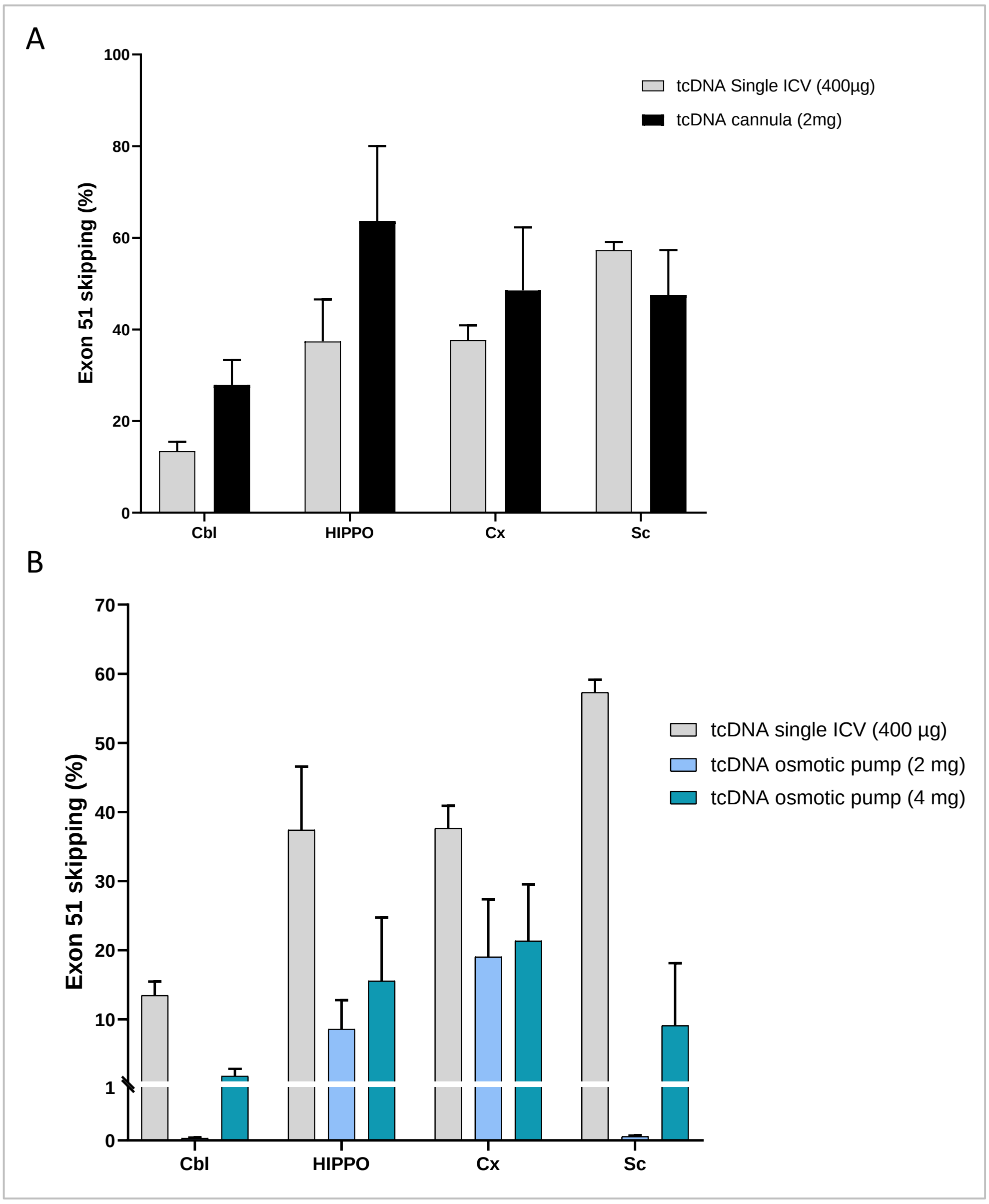

Supplementary figure 4: Comparison of single and repeated ICV delivery of tcDNA in hDMD mice. (A) Quantification of exon 51-skipping levels by RT-qPCR in different brain regions (CBL: cerebellum, CX: cortex, HIPPO: hippocampus and SC: spinal cord) after tcDNA-Ex51 repeated ICV injections via a cannula (2 mg), analyzed 4 weeks after administration. Results are expressed as means ± SEM; n=4 hDMD mice. (B) Quantification of exon 51-skipping levels by RT-qPCR in different brain regions (CBL: cerebellum, CX: cortex, HIPPO: hippocampus and SC: spinal cord) after very slow infusion of 2 and 4 mg of tcDNA-Ex51 via osmotic pumps, analyzed 4 weeks after administration. Results are expressed as means ± SEM; n=4 hDMD mice.

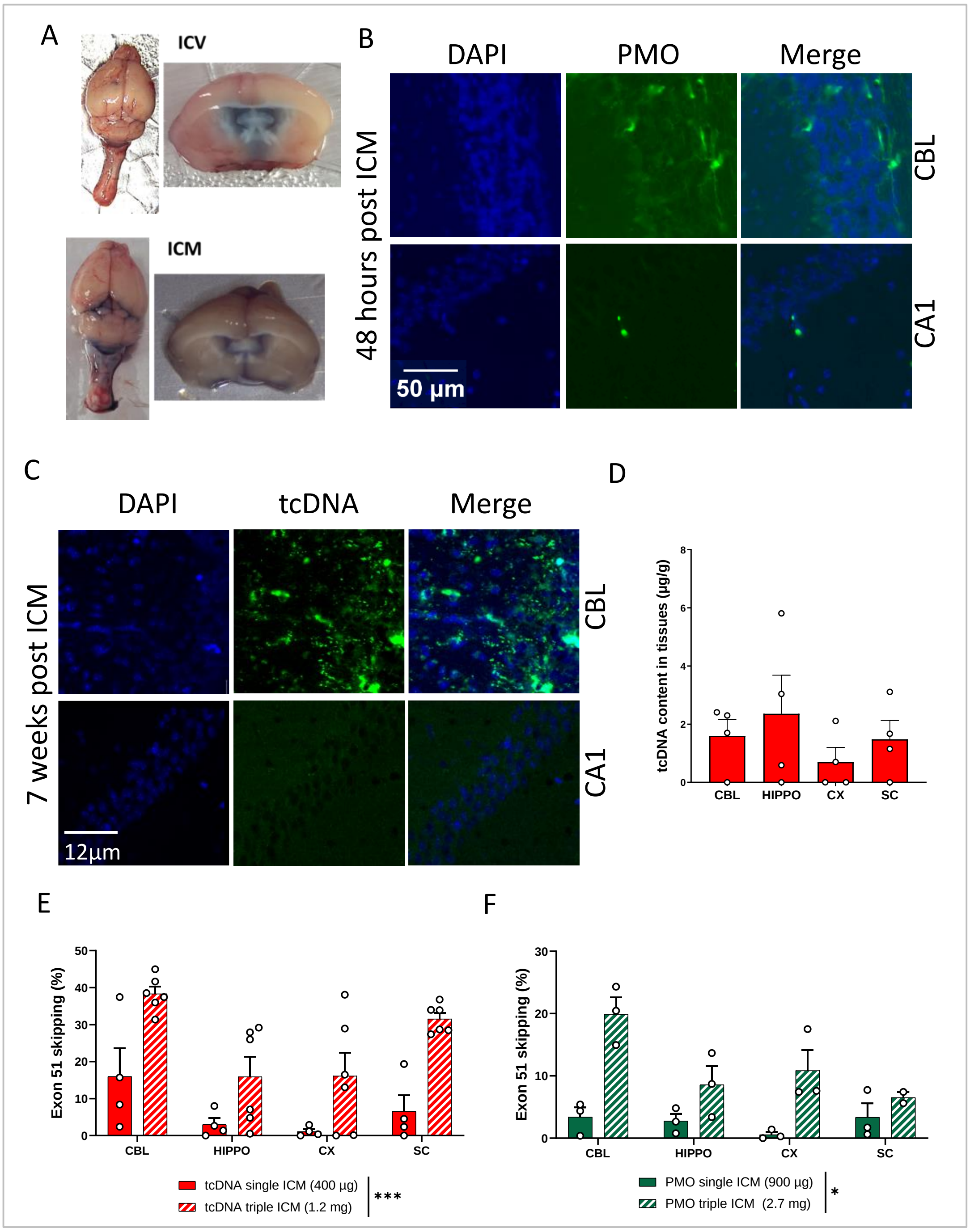

Supplementary figure 5: Biodistribution of ASO following ICM delivery. (A) Trypan blue diffusion following ICV and ICM injections. (B) Carboxyfluorescein-conjugated PMO detected 48 hours after ICV injection in the hippocampus (CA1) and cerebellum (CBL). Scale bar 50 µm. (C) Fluorescent *in situ* hybridization of tcDNA-Ex51 7 weeks after ICM injection in the CA1 of the hippocampus and the cerebellum (CBL). Scale bar 12µm. (D) Quantification by fluorescent hybridization assay of tcDNA-Ex51 content in various CNS regions (CBL: cerebellum, CX: cortex, HIPPO: hippocampus and SC: spinal cord) 7 weeks after ICM delivery. Results are expressed as means ± SEM; n=4 mdx52-tcDNA-Ex51. (E) Quantification of exon 51-skipping levels by RT-qPCR in different brain regions (CBL: cerebellum, CX: cortex, HIPPO: hippocampus and SC: spinal cord) after tcDNA single ICM compared to tcDNA triple ICM, analyzed 7 weeks after administration. Results are expressed as means ± SEM; n=4 single

ICM and n=6 triple ICM. RM Two-way ANOVA: structure effect p=0.0017, ASO chemistry effect ******* p=0.0005. (F) Quantification of exon 51-skipping levels after PMO single ICM compared to PMO triple ICM, analyzed 7 weeks after administration. Results are expressed as means ± SEM; n=3 single ICM and n=3 triple ICM. RM Two-way ANOVA: structure effect p=0.0224, ASO chemistry effect ***** p=0.0205.

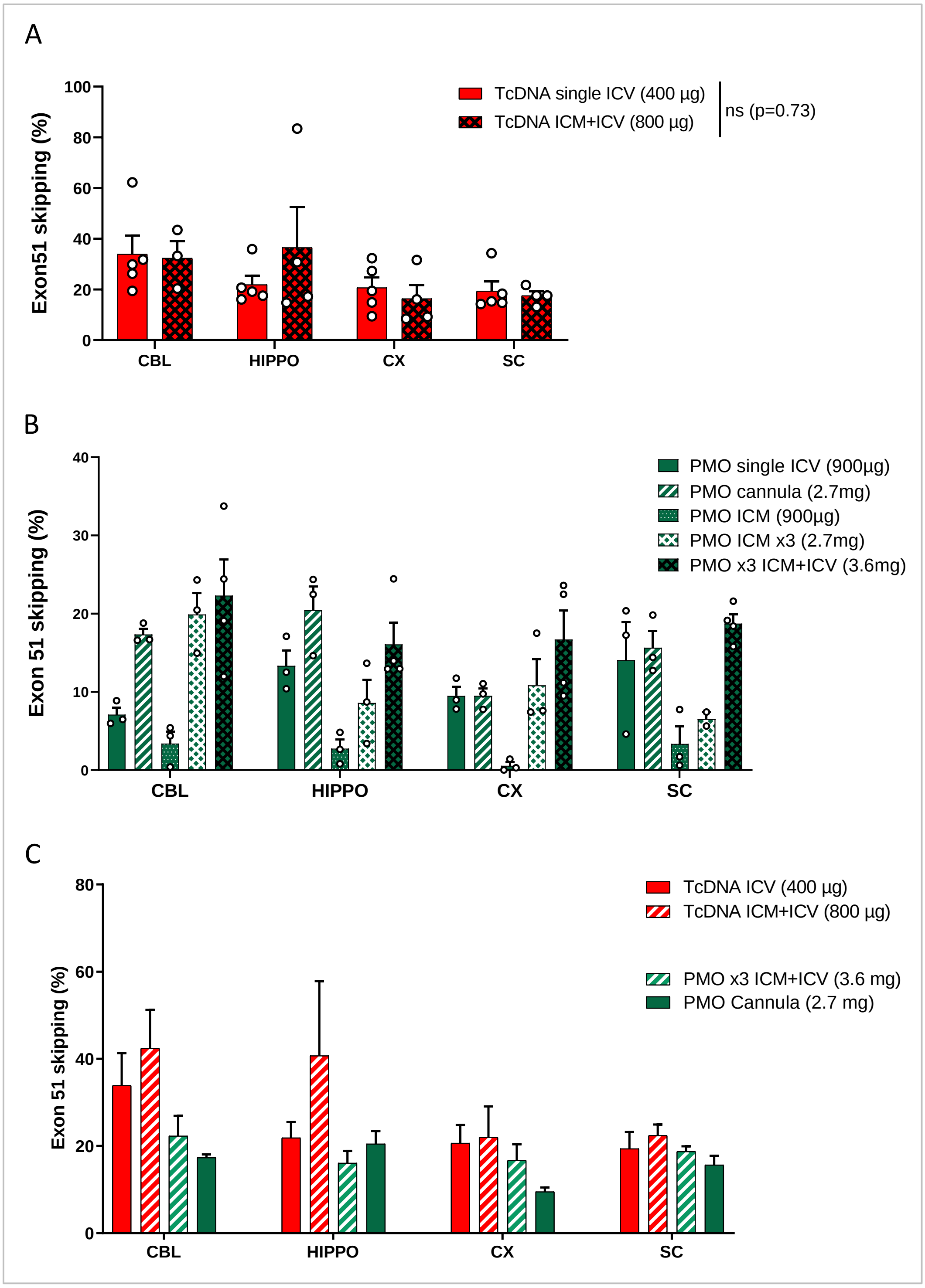

Supplementary figure 6: Comparison of various delivery routes for tcDNA and PMO. (A) Quantification of exon 51-skipping levels by RT-qPCR in different brain regions (CBL: cerebellum, CX: cortex, HIPPO: hippocampus and SC: spinal cord) after tcDNA ICM+ICV injection compared to tcDNA single ICV, analyzed 7 weeks after administration. Results are expressed as means ± SEM; n=5 single ICV and n=4 ICM+ICV. RM Two-way ANOVA: ASO chemistry effect p=0.73. (B) Comparison of exon skipping level measured in various brain regions after various delivery routes of PMO-Ex51. (C) Comparison of the 2 best delivery routes for tcDNA and PMO in various brain regions. Results are expressed as means ± SEM.

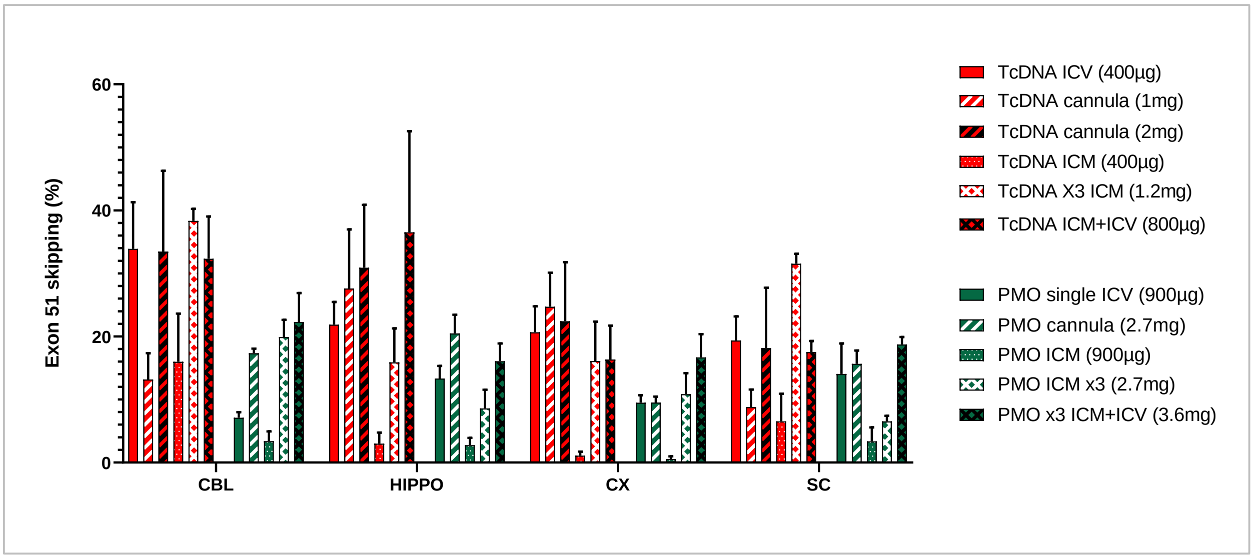

Supplementary figure 7: Summary of exon skipping efficacy following various delivery routes for tcDNA and PMO. Quantification of exon 51-skipping levels by RT-qPCR in different brain regions (CBL: cerebellum, CX: cortex, HIPPO: hippocampus and SC: spinal cord). Results are expressed as means ± SEM.
